# Dominant Heterogeneity of Upper and Lower Motor Neuron Degeneration to Motor Manifestation of Involved Region in Amyotrophic Lateral Sclerosis

**DOI:** 10.1038/s41598-019-56665-8

**Published:** 2019-12-27

**Authors:** Jiaoting Jin, Fangfang Hu, Qiuli Zhang, Qiaoyi Chen, Haining Li, Xing Qin, Rui Jia, Li Kang, Yonghui Dang, Jingxia Dang

**Affiliations:** 1 Department of Neurology, The First Affiliated Hospital of Xi’an Jiaotong University, 277 Yanta West Road, Xi’an, Shaanxi 710061 China; 2 Department of Medical Imaging, The First Affiliated Hospital of Xi’an Jiaotong University, 277 Yanta West Road, Xi’an, Shaanxi 710061 China; 3 Department of Environmental Medicine, New York University School of Medicine, New York, NY USA; 4 College of Medicine & Forensics, Xi’an Jiaotong University Health Science Center, 76 West Yanta Road, Xi’an, Shaanxi 710061 China

**Keywords:** Outcomes research, Amyotrophic lateral sclerosis

## Abstract

The aim of this study was to localize the anatomic distribution of upper motor neuron (UMN) loss through examining cortical thickness at the clinical onset of amyotrophic lateral sclerosis (ALS) and explore motor manifestation in functionally impaired body region attribute to impairment of lower motor neuron (LMN) or UMN or mixed LMN and UMN? The clinical features, cortical thickness of corresponding areas from different body regions in MRI and electromyography (EMG) data were collected from 108 classical ALS patients. The cortical thickness was thinner in ALS group than control group in bilateral head-face and upper-limb areas (p < 0.05). In head-face area, the cortical thickness of bulbar-onset group was significantly lower than that of control groups (p < 0.05). In upper-limb areas, the cortical thickness of cervical-onset group was significantly thinner than that of control group. Notably, the bulbar ALSFRS-R subscore was correlated with cortical thickness in bilateral head-face areas (p < 0.05). The bulbar ALSFRS-R subscore of the positive LMN damage group was lower compared to that of the negative LMN damage group (P < 0.001). The limb ALSFRS-R subscore correlated with compound muscle action potential (CMAP) amplitudes of median, ulnar, peroneal, and tibial nerves (P < 0.001), but was not related to cortical thickness. In conclusion, the UMN degeneration in ALS was derived from focal initiation, bulbar- and cervical-onset may date from head-face and upper-limb areas in motor homunculus cortex, respectively. The bulbar dysfunction was resulted from the mixed UMN and LMN impairment, while limb dysfunction derived mostly from LMN loss.

## Introduction

Amyotrophic lateral sclerosis (ALS) is clinically characterized by progressive dysfunction of upper motor neuron (UMN) and lower motor neuron (LMN). Motor phenotypes in ALS are widely considered to present high clinical heterogeneity and determined by 4 independent attributes: region of onset, relative mixture of UMN and LMN involvement, progression rate and spreading^1^. The most heterogeneous is motor manifestation, which is attributable to the superimposition of motor deficits occurring simultaneously at both UMN and LMN levels. But which impairment (LMN, or UMN, or mixed LMN and UMN) result in motor manifestation in functionally impaired body region? What about the dominance of UMN and LMN degeneration to motor manifestation of involved region in ALS. So far, no relevant literature has been reported. To decipher the relationship between the motor manifestation and UMN or LMN deficit, the objective and reliable UMN and LMN deficit marker in ALS was highly desirable. The measurement of motor cortical thickness appears to be a useful and sensitive method for UMN impairment in ALS^2–4^. Several surface-based MRI morphometry studies have demonstrated significant thinning of the motor cortex in ALS patients with the potential to track ALS spread along the motor cortex^3–6^. Schuster *et al*. reported that bulbar-onset patients showed cortical thinning in the bilateral bulbar-segment of motor homunculus. In contrast, patients with lower limb-onset demonstrated thinning in the bilateral segments of the motor homunculus representing arms and legs^3^. The different region onset was an aspect of clinical heterogeneity in ALS^1^. It has been suggested that different body onsets correspond to different motor homunculus partitions^3^. Therefore, when localizing the anatomic distribution of UMN loss, more detailed motor partitions are bound to help characterize UMN damages. Recently, Jiang *et al*. proposed a more detailed and refined division of the motor homunculus cortex, which comprised of head-face, tongue-larynx, upper-limb, trunk, and lower-limb areas^7^.

Objective methodologies to detect LMN injury have been sophisticated by neurophysiological techniques, with conventional electromyography (EMG) being the most developed and refined^1^. Specifically, the positive sharp waves (PSW) and fibrillation or fasciculation potential could indicate whether LMN was involved^8,9^. Additionally, compound muscle action potential amplitude (CMAP) can reflect the degree of unique LMN involvement^10,11^. The functionally impaired body regions can also be easily detected by EMG^12^.

The Amyotrophic Lateral Sclerosis Functional Rating Scale revised version (ALSFRS-R), which is employed to assess the disability degree of motor manifestation, has been used as a primary outcome measure in clinical trial^13,14^. It is considered clinically meaningful and could predict survival time^15^. Multicenter clinical trials using ALSFRS-R could more easily implement outside specialized ALS centers because it do not require specialized instrument. The ALSFRS-R has shown excellent inter- and intra-rater reliability, and reliability of telephone administration when used as primary outcome measure in a multicenter ALS trial, even more, it is reliable when administered to the caregiver^16–18^.

The aim of our study was to localize the anatomic distribution of UMN loss through examining cortical thickness at the clinical onset of ALS and explore motor manifestation in functionally impaired body region attribute to impairment of LMN or UMN or mixed LMN and UMN?

## Materials and Methods

### Participants

According to the revised El Escorial criteria^19^, ALS patients were recruited from ALS Clinics of the First Affiliated Hospital of Medical College of Xi’an Jiaotong University between January 2014 and December 2016. Classical ALS patients with simultaneous signs of UMN and LMN involvement in one or more regions were included in our study cohorts. Non-classical patients with only signs of UMN or LMN involvement, for instance primary lateral sclerosis, progressive muscular atrophy, flail arm, or flail leg syndrome, were not included. All recruited ALS patients were right-handed. The control participants were healthy volunteers, including patient’s spouse, siblings, or age-matched persons living in the same environment with the patients. All participants with a history of hypertension, diabetes, stroke, migraine, frontotemporal dementia, cerebrovascular events, psychiatric disease, and other neurological diseases were excluded. We confirmed that informed consent was obtained from all participants. Our study was approved by the Research Ethics Committee of the First Affiliated Hospital of Medical College of Xi’an Jiaotong University. We confirmed that our study was performed in accordance with relevant guidelines and regulations.

### Clinical data

All patients underwent the same diagnostic procedures, including clinical examination, electrodiagnostic examination, laboratory blood testing, as well as brain and cervical spinal cord MRI to exclude other diseases resembling ALS. Patients were classified based on site-onset, including bulbar, cervical, and lumbosacral. Disease duration was defined as the interval from the onset of symptoms to the first visit. All patients were classified as definite, probable, probable-lab supported, or possible ALS according to diagnostic criteria. Possible ALS patients have progressed to definite or probable diagnosis at the follow-up study. The ALSFRS-R was a disease-specific functional rating scale. Subgroups of bulbar (items 1–3), upper limbs (items 4–6) and lower limbs (items 7–9) were covered in ALSFRS-R. The maximum score of each subgroup was 12. There were no significant differences in age and gender between ALS patients and control groups (p > 0.05). Demographic and clinical characteristics were listed in Table 1.

**Table 1:** Demographic and clinical characteristics of study participants.

	ALS group	Control group
Number	108	90
Age, median (SD)	52.83(9.49)	51.63 (9.68)
Gender (Male/Female)	65/43	46/44
**Site of onset (%)**		
Bulbar	13 (12.0)	
Cervical	74 (68.5)	
Lumbosacral	21 (19.4)	
ALSFRS-R score, median (SD)	39.3 (5.87)	
Bulbar ALSFRS-R score	10.7 (2.02)	
Upper limb ALSFRS-R score	7.7 (3.27)	
Lower limb ALSFRS-R score	9.1 (2.73)	
Disease duration in months, median (SD)	14.69 (11.98)	
**El Escorial score (%)**		
Definite	20 (18.5)	
Probable	58 (53.7)	
Probable-lab supported	20 (18.5)	
Possible	10 (9.3)	

### MRI data

T1WI images were performed on 3.0T MRI scan (GE signal HDxt) using 8-channel head coil. TR = 10.8 ms, TE = 2.98 ms, flip angle = 9, FOV = 256 mm × 256 mm, matrix = 256 × 256, voxel = 1 mm × 1 mm × 1 mm, thickness = 1 mm, no gap. Conventional T2WI images were also carried out in order to rule out patients with possible brain damages. The cortical thickness of each subject was measured by employing the FreeSurfer programe (version 6.0). The tissue segmentation confirmed the boundaries between white/grey matter (white matter surface) and grey matter/cerebrospinal fluid (pial surface). Cortical thickness was defined as the distance from the white matter surface to the nearest point on the pial surface. The visual evaluation of cortical thickness was performed by an experienced radiologist who had 10 years of MR imaging reporting experience and was blinded of the condition of the participants. The regions of interest (ROIs) of motor homunculus cortex were selected on the basis of Jiang’s cortex partition^7^.

### Electrodiagnostic examination data

The electrodiagnostic examinations were carried out using standard electromyography instrument (EDX EMG/evoked potential equipment, Nicolet, America). All patients were examined by a neurophysiologist with more than 15 years of electrodiagnostic experience. The nerve conduction studies were performed using standard procedures. The CMAP of bilateral motor nerves (median, ulnar, peroneal and tibial) were assessed. The bilateral muscles of bulbar were detected by needle EMG, including sternocleidomastoid, tongue, and lower orbicularis oris^12^. The presence of PSW, fibrillation, or fasciculation potential which were recommended in the Awaji criteria of ALS^20^ in the muscles of body region was considered to indicate LMN damage, and regarded as the positive LMN damage group. On the contrary, the absence of that was considered as the negative LMN damage group.

### Statistical analysis

Normal distribution of data was assessed using the Kolmogorov-Smirnov test. Significant group differences in age and gender were identified via two-sample t-tests and Pearson’s chi-square test in ALS and control groups, respectively. The general multivariate linear model was performed to compare corresponding ROI cortical thickness between ALS and control groups, between bulbar, cervical, lumbosacral onset and control groups, considering total intracranial volume (TIV) and age as covariates. All analyses were Bonferroni-corrected for multiple comparisons. Bivariate correlation was used to analyze the correlation of bulbar, upper-limb, and lower-limb ALSFRS-R scores with the residuum of corresponding ROI cortical thickness, the correlation of upper-limb ALSFRS-R score with the CMAP of median and ulnar nerves, and lower-limb ALSFRS-R score with peroneal and tibial nerves. The two-sample t-test was used to compare the bulbar ALSFRS-R score between positive LMN damage and negative LMN damage groups in bulbar region. P-values below 0.05 were considered statistically significant.

## Results

### Differences of ROI cortical thickness between ALS and control groups

**Figure 1:**
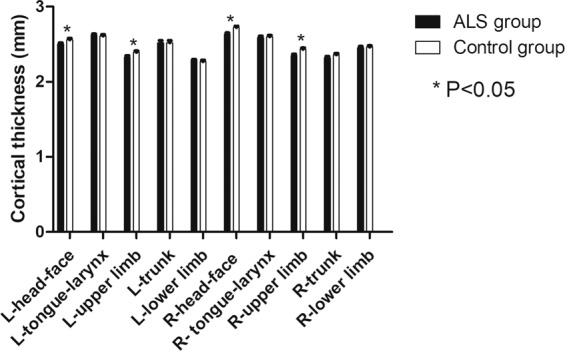
Differences of ROIs cortical thickness between ALS and control groups.

Cortical thickness was significantly thinner in ALS group compared to control group in bilateral head-face and upper-limb areas (L-head-face p = 0.017, R-head-face p = 0.032, L-upper-limb p = 0.0005, R-upper-limb p = 0.013, Fig. 1). However, thinning in bilateral tongue-larynx, trunk, and lower-limb areas in motor homunculus was not found (p > 0.05; Fig. 1).

### Differences of ROIs cortical thickness between bulbar-, cervical-, lumbosacral-onset and control groups

**Figure 2:**
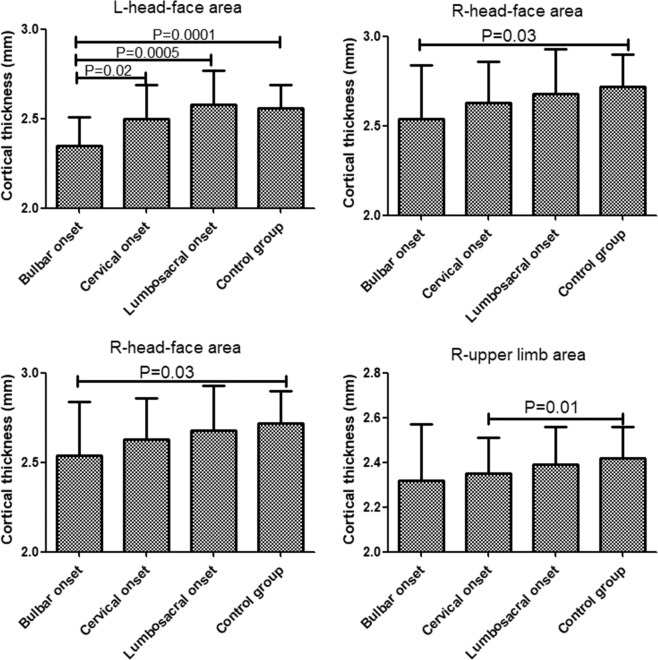
Differences of ROIs cortical thickness between bulbar-, cervical-, lumbosacral-onset and control groups.

There were statistically significant differences between bulbar-, cervical-, lumbosacral-onset and control groups in bilateral head-face and upper-limb areas (p < 0.01; Fig. 2, Table 2). However, no statistical differences were observed in bilateral tongue-larynx, trunk, and lower-limb areas in motor homunculus (p > 0.05; Table 2). Through pairwise comparisons, in left head-face area, the cortical thickness of bulbar-onset group was significantly lower than that of cervical-onset, lumbosacral-onset, and control groups (p < 0.05; Fig. 2); whereas in right head-face area, the cortical thickness of bulbar-onset group was only significantly less than that of healthy control group (p < 0.05; Fig. 2). In regard to bilateral upper-limb areas, the cortical thickness of cervical-onset group was significantly thinner than that of control group (p < 0.05; Fig. 2).

**Table 2:** Differences of ROI cortical thickness between bulbar, cervical, lumbosacral onset and control groups.

ROI	Bulbar-onset ($\bar{{\rm{X}}}$ ± SD)mm	Cervical-onset ($\bar{{\rm{X}}}$ ± SD)mm	Lumbosacral-onset ($\bar{{\rm{X}}}$ ± SD)mm	Control group ($\bar{{\rm{X}}}$ ± SD)mm	GLM main effect	
	(n = 13)	(n = 74)	(n = 21)	(n = 90)	F	P
L-head-face	2.35 ± 0.16	2.50 ± 0.19	2.58 ± 0.19	2.56 ± 0.13	7.93	0.00005^*^
L-tongue-larynx	2.68 ± 0.15	2.62 ± 0.16	2.62 ± 0.19	2.60 ± 0.17	0.68	0.57
L-upper limb	2.28 ± 0.25	2.32 ± 0.18	2.40 ± 0.18	2.39 ± 0.14	5.21	0.002^*^
L-trunk	2.64 ± 0.26	2.50 ± 0.29	2.57 ± 0.34	2.51 ± 0.30	1.01	0.39
L-lower limb	2.27 ± 0.13	2.28 ± 0.15	2.31 ± 0.17	2.27 ± 0.14	1.47	0.23
R-head-face	2.54 ± 0.30	2.63 ± 0.23	2.68 ± 0.25	2.72 ± 0.18	3.99	0.01^*^
R-tongue-larynx	2.59 ± 0.18	2.60 ± 0.16	2.54 ± 0.23	2.59 ± 0.16	0.60	0.61
R-upper limb	2.32 ± 0.25	2.35 ± 0.16	2.39 ± 0.17	2.42 ± 0.14	4.20	0.01^*^
R-trunk	2.29 ± 0.21	2.32 ± 0.18	2.32 ± 0.17	2.36 ± 0.18	1.15	0.33
R-lower limb	2.46 ± 0.17	2.44 ± 0.17	2.50 ± 0.20	2.46 ± 0.16	0.92	0.43

^*^p < 0.05, see table e-1 for post hoc GLM p-values comparing bulbar, cervical, lumbosacral onset and control groups.

### Correlations among ROIs in motor homunculus

**Figure 3:**
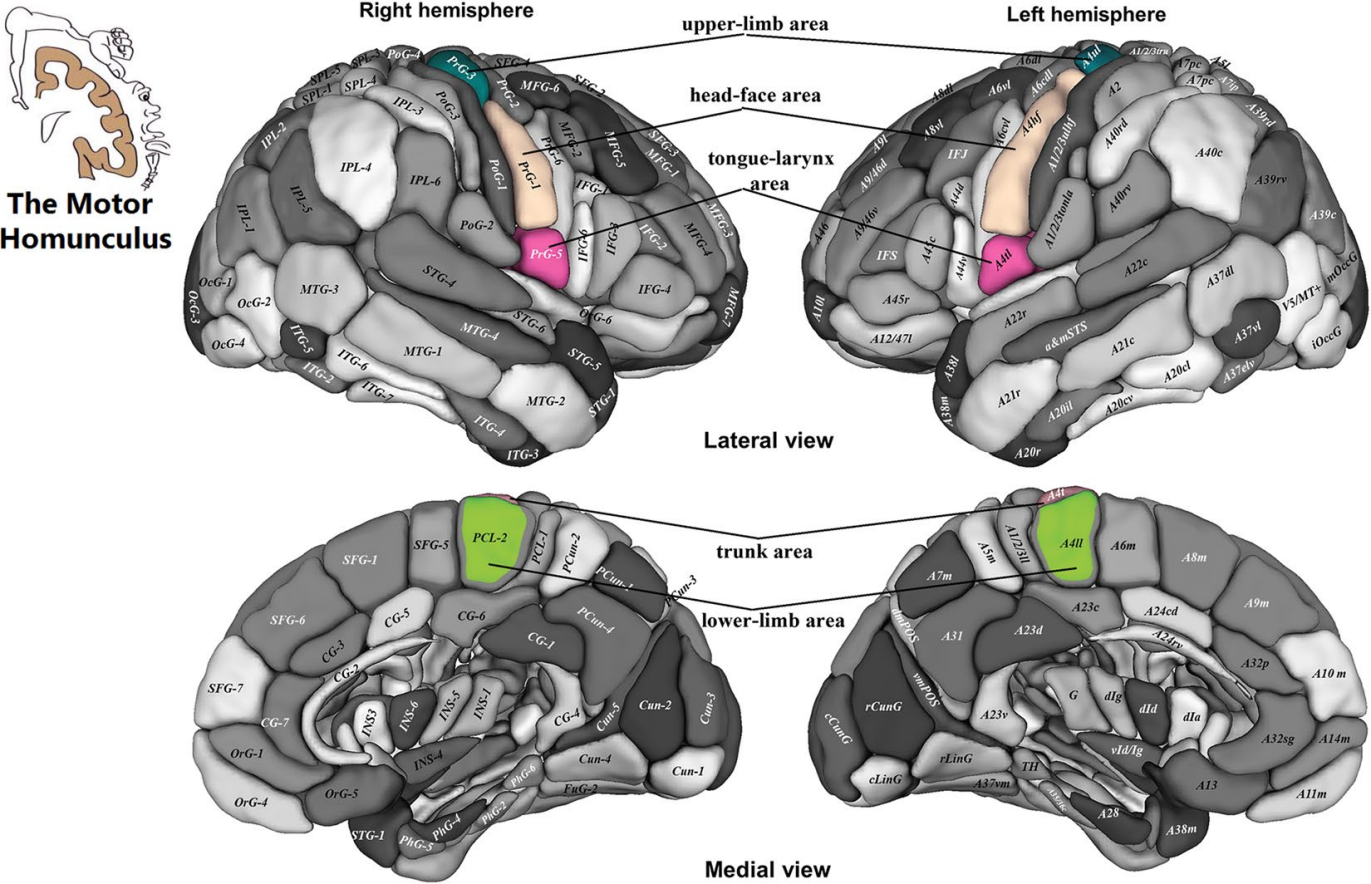
ROIs in the motor homunculus, the tongue-larynx area corresponding to the PrG5, the head-face area corresponding to PrG-1, the upper-limb area corresponding to PrG-3, the trunk area corresponding to PrG-4, and the lower-limb area corresponding to PCL-2. PrG: Precentral gyrus, PCL: Paracentral lobule Note: This figure is modified from part A and B in the Fig. 2 on Tianzi Jiang’s article. Modifications to the original figure including tongue-larynx, head-face, upper-limb, trunk, and lower-limb with the corresponding regions^7^.

In left motor homunculus, the tongue-larynx area was not correlated with trunk and lower-limb area, but beyond that, there was a significant statistical correlation between any two ROIs (Table 3, Fig. 3). In right motor homunculus, the tongue-larynx area was not correlated with upper-limb, trunk, and lower-limb area, but beyond that, there was a significant statistical correlation between any two ROIs (Table 4, Fig. 3).

**Table 3:** Correlations among ROIs in left motor homunculus cortex.

	Tongue-larynx		Head-face		Upper-limb		Trunk		Lower-limb	
	C	P	C	P	C	P	C	P	C	P
Tongue- larynx	—	—								
Head- face	0.21	0.03^*^	—	—						
Upper- limb	0.22	0.02^*^	0.74	3.14 × 10^−20*^	—	—				
Trunk	0.09	0.37	0.38	5.0 × 10^−5*^	0.46	7.56 × 10^−7*^	—	—		
Lower- limb	0.03	0.77	0.55	1.07 × 10^−9*^	0.60	7.77 × 10^−12*^	0.42	6.0 × 10^−6*^	—	—

^*^p < 0.05; C: correlation coefficient.

**Table 4:** Correlations among ROIs in right motor homunculus cortex.

	Tongue-larynx		Head-face		Upper-limb		Trunk		Lower-limb	
	C	P	C	P	C	P	C	P	C	P
Tongue- larynx	—	—								
Head- face	0.23	0.02^*^	—	—						
Upper- limb	0.03	0.74	0.57	1.77 × 10^−10*^	—	—				
Trunk	0.14	0.15	0.46	5.04 × 10^−7*^	0.60	5.87 × 10^−12*^	—	—		
Lower- limb	0.16	0.09	0.44	2.0 × 10^−6*^	0.63	3.85 × 10^−13*^	0.63	4.0 × 10^−13*^	—	—

^*^p < 0.05; C: correlation coefficient.

### Relationship between ALSFRS-R subscore and the residuum of corresponding ROIs cortical thickness, and electrodiagnostic examination data

**Figure 4:**
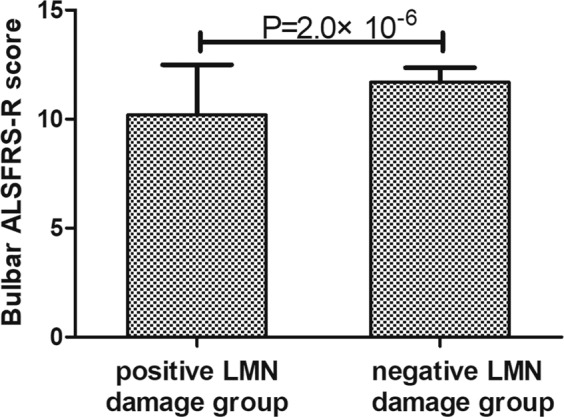
Differences of bulbar ALSFRS-R subscore between positive LMN damage and negative LMN damage groups.

The bulbar ALSFRS-R subscore was correlated with the residuals of cortical thickness in bilateral head-face areas (Left p = 3.89 × 10^−4^, r = 0.34; Right p = 0.002, r = 0.29), and uncorrelated with the residuals of cortical thickness in bilateral tongue-larynx areas (p > 0.05). The bulbar ALSFRS-R subscore of positive LMN damage group was significantly lower than that of negative LMN damage group (p = 2.0 × 10^−6^, Fig. 4). The upper-limb ALSFRS-R score was not related with the residuals of cortical thickness in bilateral upper-limb areas (p > 0.05), but was related to CMAP of median (p = 3.42 × 10^−7^; r = 0.49) and ulnar nerves (p = 3.4 × 10^−5^; r = 0.40). The lower-limb ALSFRS-R score showed no correlation with the residuals of cortical thickness in bilateral lower-limb areas (p > 0.05), but showed significant correlation with CMAP of peroneal (p = 0.001; r = 0.34) and tibial nerves (p = 8.3 × 10^−5^; r = 0.38).

## Discussion

In this study, motor neuron degeneration in UMN and LMN levels was detected through examining cortical thickness and EMG in ALS patients, respectively. We found that bulbar-onset patients manifested cortex thinning of head-face areas and bulbar denervation in EMG, both of them were related to bulbar ALSFRS-R subscore. Although the cervical-onset patients showed atrophy of upper-limb areas, the motor dysfunction was mainly related to LMN impairment, just like lumbosacral-onset patients. The results suggested the dominance heterogeneity of UMN and LMN in patients with different onset of ALS.

In our study, we found that the motor homunculus cortex thinning was focal, rather than globally. Specifically, the bilateral head-face and upper-limb areas were significantly thinner in patients with ALS than controls. Lots of studies have indicated that motor cortex atrophy in ALS patients were caused by motor neuron loss^2,3,6^, which were largely induced by environmental, infectious, or metabolic factors, as well as toxicity of free radicals, protein aggregates, apoptosis, and mutations of superoxide dismutase 1 (SOD1)^21–24^. Swash pointed out that interactions among multiple risk factors determined the relative susceptibility and resistance for generalized abnormality^25^, thus resulting in focal impairment in motor homunculus cortex. So the UMN degeneration in ALS was derived from focal initiation.

Another important finding was that cortical thickness differed in their site of disease onset in terms of bulbar, cervical and lumbosacral onset. Patients with bulbar- and cervical-onset revealed the most pronounced thinning in the bilateral head-face and upper-limb areas of motor cortex. However, patients with lumbosacral-onset failed to display significant cortical thinning in the motor homunculus cortex. The reasons for this may be complex. Firstly, from the physiological level, the absence of monosynaptic connections between the motor cortex and lumbosacral neuron might impact neurotransmitter transport in UMN and LMN, even the lumbosacral denervation was pronounced in EMG. While monosynaptic connection was an important connection pattern in the motor cortex and lower motor neurons at bulbar and cervical level. Secondly, this might be in part associated with the anatomical characteristic in the lower-limb motor cortex. The Betz cells in the motor cortex were not equally distributed. The total number of Betz cells is 25,000–34,000, approximate 75% of Betz cell located in the lower-limbs areas, while 17.9% and 6.6% of them were distributed in the upper-limbs and head-face areas^26^. Betz cell loss in the motor cortex has been widely detected in patients with ALS^1–3,5,6^. While in our study, we suggested that even the absolute Betz cells loss were comparable in different anatomical regions of the motor cortex in ALS patients, it might be not sensitive enough for the freesurfer program to detect significant cortical thinning in the lower-limb regions, in contrast with upper-limb and head-face areas. Thirdly, the more functional importance of upper-limb and head-face areas in motor homunculus cortex was another important reason. Compared with lower-limb areas, the function of head-face and upper-limb areas are more anatomical-physiological important and exquisite, also more energy costly. The high need of energy in the head-face and upper-limb areas made them more vulnerable to excitotoxicity and oxidative stress^21^. So the UMN degeneration of bulbar- and cervical-onset may date from head-face and upper-limb areas in motor homunculus cortex, respectively.

Correlations among ROIs in motor homunculus cortex have showed that the tendency of correlation was related to the differing distances of their anatomic spans, the closer the distance, the easier they might be correlated, and vice versa. Our results might be consistent with that UMN spread contiguously outward along the motor somatotopic homunculus cortex^27^. In our study, we found that the motor homunculus cortex thinning in bilateral head-face and upper-limb area. ROIs shrink synchronously are more likely to show numerical correlation. Otherwise, it may manifest as numerical irrelevance. Our conclusion was supported by phosphorylated TDP-43 pathology and neuroradiological evidences. A model of staging of pTDP-43 pathology in ALS has been recently proposed, pathology may originate in the primary motor cortex, and subsequently spreads to other areas of the cerebral cortex and to the connected subcortical structures^28^. The TDP-43 could be transmitted from neuron to neuron via synapses between axon and soma^29^. In neuroradiology, structural and functional connectivity are more impaired in regions that are topologically more proximate to the motor cortex relative to more remote ones^30^ and structural connectivity undergoes a progressive impairment^31^.

Furthermore, in order to illuminate the relationship between motor dysfunction and impairment of LMN or UMN or mixed LMN and UMN, we analyzed the correlation between the ROI cortical thickness, electrodiagnostic examination, and ALSFRS-R subscores. Our results implied that bulbar dysfunction scored by ALSFRS-R resulted from the mixed involvement of UMN and LMN, while limb dysfunction was primarily generated from LMN impairment. The most likely reason may be the relationship between UMN and LMN in bulbar and spinal region. Ravits showed that the degeneration of UMN and LMN spread contiguously and independently through the motor system’s 3-dimensional^1,27^. The anatomical distance from motor cortex to bulbar region (corticobulbar tract) was shorter than the distance from motor cortex to spinal body region (corticospinal tract), so correlation between UMN and LMN involvement in the bulbar region was more easily emerged than in the spinal region.

A major limitation of this cross-sectional study is the lack of available follow-up data. Longitudinal data may help clarify the sequential order of clinically measurable changes and morphological alterations in motor cortex. Another limitation of this study is the number of bulbar patients was small, the reason as follow, first, only 14% had the bulbar-onset form in Chinese ALS population^32^. Second, the patients with bulbar-onset were older than limb-onset, had more concomitant diseases, so partial patients were eliminated in participant recruitment. Third, some patients with bulbar-onset couldn’t tolerate long time MRI scanning. Additionally, the ALSFRS-R measured the extent to which ALS patients were capable of performing functional activities. It was more about assessing the dysfunction of LMN impairment, so the correlations of ALSFRS-R and LMN loss were more easily been detected.

In conclusion, the UMN degeneration in ALS was derived from focal initiation, bulbar- and cervical-onset may date from head-face and upper-limb areas in motor homunculus cortex, respectively. The bulbar dysfunction was resulted from the mixed UMN and LMN impairment, while limb dysfunction derived mostly from LMN loss.

## Supplementary information

Supplementary information.

## Data Availability

The datasets generated and analysed during the current study are available from the corresponding author on reasonable request.
