# Supplementary material for: Dominant Heterogeneity of Upper and Lower Motor Neuron Degeneration to Motor Manifestation of Involved Region in Amyotrophic Lateral Sclerosis
Source: Sci Rep. 2019 Dec 27;9:20059. doi: 10.1038/s41598-019-56665-8 (PMC6934517; doi:10.1038/s41598-019-56665-8)
Supplement: Supplementary file 1 — Supplementary information. [file 41598_2019_56665_MOESM1_ESM.pdf]

Dominant Heterogeneity of Upper and Lower Motor Neuron Degeneration to Motor Manifestation of Involved Region in Amyotrophic Lateral Sclerosis

Jiaoting Jin*[1]; Fangfang Hu*[1]; Qiuli Zhang[2]; Qiaoyi Chen[3]; Haining Li[2]; Xing Qin[1]; Rui Jia[1]; Li Kang[1]; Yonghui Dang[§4] & Jingxia Dang[§1]

* Both authors contributed equally

[1] Department of Neurology, The First Affiliated Hospital of Xi'an Jiaotong University, 277 Yanta West Road, Xi'an, Shaanxi710061, China

[2] Department of Medical Imaging, The First Affiliated Hospital of Xi'an Jiaotong University, 277 Yanta West Road, Xi'an, Shaanxi 710061, China

[3] Department of Environmental Medicine, New York University School of Medicine, New York, NY, USA

[4] College of Medicine & Forensics, Xi'an Jiaotong University Health Science Center, 76 West Yanta Road, Xi'an, Shaanxi 710061, China

[§] Corresponding authors

Corresponding author contact:

1.Yonghui Dang, Professor

College of Medicine & Forensics, Xi'an Jiaotong University Health Science Center

76 West Yanta Road, Xi'an, Shaanxi, 710061, China

Email: psydyh@mail.xjtu.edu.cn

2. Jingxia Dang, Professor

Department of Neurology. The First Affiliated Hospital, Medical College, Xi'an Jiaotong University

277 Yanta West Road, Xi'an, Shaanxi, 710061 ，China

Telephone number: +86 13379262486

Email: jxdang2000@126.com

Author's email

Jiaoting Jin:jinjiaoting@qq.com

Fangfang Hu:hufang2008tree@163.com

Qiuli Zhang:akireizhang@gmail.com

QiaoyiChen:qyc203@nyu.edu

Haining Li:li9717@126.com

Xing Qin:thornbird2008@163.com

RuiJia:jiarui029@163.com

Li Kang:bemyselfconnie@sina.com

Yonghui Dang:psydyh@mail.xjtu.edu.cn

Jingxia Dang: jxdang2000@126.com

Table e-1 P-valuesfor pairwise comparisonsbetween bulbar, cervical, lumbosacral onset group and control group

	$P_{1vs\ 2}$	P_{1vs3}	P_{1vs4}	P_{2vs3}	P_{2vs4}	P_{3vs4}
L-head-face	0.02	0.0005	0.0001	0.22	0.09	1.00
L-tongue-larynx	1.00	1.00	0.94	1.00	1.00	1.00
L-upper limb	1.00	0.11	0.08	0.10	0.01	1.00
L-trunk	0.76	1.00	1.00	1.00	1.00	1.00
L-lower limb	1.00	0.52	0.59	1.00	1.00	1.00
R-head-face	1.00	0.42	0.03	1.00	0.05	1.00
R-tongue-larynx	1.00	1.00	1.00	1.00	1.00	1.00
R-upper limb	1.00	0.96	0.16	1.00	0.01	1.00
R-trunk	1.00	1.00	1.00	1.00	0.81	1.00
R-lower limb	1.00	1.00	1.00	0.87	1.00	1.00

1: bulbar onsetgroup, 2:cervical onset group, 3:lumbosacral onset group, 4:controlgroup
